# Supplementary material for: Protecting the public interest while regulating health professionals providing virtual care: A scoping review
Source: PLOS Digit Health. 2023 Apr 28;2(4):e0000163. doi: 10.1371/journal.pdig.0000163 (PMC10146454; doi:10.1371/journal.pdig.0000163)
Supplement: Attachment — Submitted filename: Review of PDIG.pdf [file pdig.0000163.s003.pdf]

Review of PDIG-D-22-00332 "Protecting the public interest while regulating health professionals providing virtual care: A scoping review"

This is an interesting piece that provides a well done synopsis of the literature on the regulation of virtual care.

The methods are clearly explained and I see no issues worth of noting with the strategy used for synthesizing the literature and presenting the results.

I have two comments to help better frame the article and perhaps make it more accessible and informative for others that are interested in the topic.

1) More acknowledgement of the potential cost of regulation

It is clear that the intent of the article from the title and focus is on the public interest aspect of regulation.

In the social sciences literature (economics and sociology, for example) there is a competing and more generally accepted view—particularly when it comes to regulation like occupational licensing—that there is a clearly defined “private interest” hypothesis.

In other words, licensing boards and regulators (that are generally dominated by market participants) may be acting in their own self interest rather than the public interest.

The authors do not ignore this view—they have some discussion of this on P.15-17, but I think the manuscript would be improved by spending more time in the intro making clear that there are competing theories of the primary driver of regulation.

Some possible citations would include:

Stigler (1971) The Theory of Economic Regulation

Friedman (1962) Capitalism and Freedom

Peltzman (1976) Toward a More General Theory of Regulation

2) Some discussion/separation of the different types of regulation

Regulation can come in many different varieties and types. First with respect to occupational regulation, it can be separated into 3 distinct types:

a) Licensing- makes it illegal to do a job without meeting minimum entry standards
b) Certification- title protection (e.g., cannot use the title “certified massage therapist” without meeting minimum entry standards)
c) Registration- government maintains a list of providers

Second—the question of scope of practice can be viewed in a separate space. Often, other providers are dictating practice boundaries. As an example, physicians may be deciding whether or not nurses can work to the full extent of their training. These types of regulations are quite different from

typical occupational regulation. There is growing evidence that SOP restrictions are not in the public interest whatsoever and are merely anticompetitive in nature. For example, see:

Adams and Markowitz (2018) Improving efficiency in the health-care system: Removing anticompetitive barriers for advanced practice registered nurses and physician assistants

Bae and Timmons (2022) Restrictions on Health Care Profession Scope of Practice: Do They Help or Harm Patients?

I think there should be some acknowledgement/clarification that there are notable differences in the types of occupational regulation used (licensing being most prevalent for healthcare occupations)

The SOP question is distinct, much more likely to be anticompetitive (private interest motivated), and not in the public interest.
