# Supplementary material for: Protecting the public interest while regulating health professionals providing virtual care: A scoping review
Source: PLOS Digit Health. 2023 Apr 28;2(4):e0000163. doi: 10.1371/journal.pdig.0000163 (PMC10146454; doi:10.1371/journal.pdig.0000163)
Supplement: Attachment — Submitted filename: PLOS Digital Health - Scoping Review Response to Reviewers.docx [file pdig.0000163.s004.docx]

**We sincerely thank the reviewers for their constructive suggestions to improve our manuscript. We have highlighted our revisions in yellow in the resubmitted manuscript. We have also included our responses to these suggestions below in red.**

**REVIEWER #1:**

I have two recommendations regarding the framing/presentation of the results to make them more accessible and also so that they better conform with the existing literature.

1. More acknowledgement of the potential cost of regulation. It is clear that the intent of the article from the title and focus is on the public interest aspect of regulation. In the social sciences literature (economics and sociology, for example) there is a competing and more generally accepted view—particularly when it comes to regulation like occupational licensing—that there is a clearly defined “private interest” hypothesis. In other words, licensing boards and regulators (that are generally dominated by market participants) may be acting in their own self interest rather than the public interest.

The authors do not ignore this view—they have some discussion of this on P.15-17, but I think the manuscript would be improved by spending more time in the intro making clear that there are competing theories of the primary driver of regulation.

Some possible citations would include: Stigler (1971) The Theory of Economic Regulation; Friedman (1962) Capitalism and Freedom; Peltzman (1976) Toward a More General Theory of Regulation.

AUTHOR RESPONSE (AU): We agree that the competing theories of regulation could be more clearly stated in the introduction. We have revised the text per your suggestion, with additions to the introduction highlighted on lines 79-85.

2. Some discussion/separation of the different types of regulation. Regulation can come in many different varieties and types. First with respect to occupational regulation, it can be separated into 3 distinct types:

a) Licensing- makes it illegal to do a job without meeting minimum entry standards

b) Certification- title protection (e.g., cannot use the title “certified massage therapist” without meeting minimum entry standards)

c) Registration- government maintains a list of providers

Second—the question of scope of practice can be viewed in a separate space. Often, other providers are dictating practice boundaries. As an example, physicians may be deciding whether or not nurses can work to the full extent of their training. These types of regulations are quite different from typical occupational regulation. There is growing evidence that SOP restrictions are not in the public interest whatsoever and are merely anticompetitive in nature. For example, see:

Adams and Markowitz (2018) Improving efficiency in the health-care system: Removing anticompetitive barriers for advanced practice registered nurses and physician assistants

Bae and Timmons (2022) Restrictions on Health Care Profession Scope of Practice: Do They Help or Harm Patients?

I think there should be some acknowledgement/clarification that there are notable differences in the types of occupational regulation used (licensing being most prevalent for healthcare occupations). The SOP question is distinct, much more likely to be anticompetitive (private interest motivated), and not in the public interest.

AU: Thank you very much for these suggestions. We agree that this review (and regulatory research more generally) is constrained somewhat by a lack of a globally accepted taxonomy and standardized terminology. We have tried to clarify this first point around different types of regulation in the introduction (lines 69-75) and under our inclusion criteria (lines 144-145). For scope of practice, this point is very important and we have now included reference to this on lines 84-85.

**REVIEWER #2:**

Thank you for the opportunity to review this interesting manuscript. It is well written and original and of interest to the community of regulators and regulated and those that study these areas. I have a few minor comments that can be considered as nuancing the paper to enhance its findings rather than any major critique.

1. At line 46 the authors state "Professional regulators are legally obligated to protect the public interest, but how the public interest is defined is amorphous and subject to social change". I of course agree with this but in light of future discussion in the paper and the intent to discuss literature from across a variety of jurisdictions I suggest also noting in this sentence that what constitutes public interest in regulation is also affected by the state/province, country or supra national entity in which regulation takes place. See for example Feintuck The Public interest in Regulation (OUP). Given the range of countries included stretch from Russia to the US this is important to clarify.

AU: We appreciate the suggestion and have revised the text to reflect your point (see lines 59-61).

2. Line 56 discusses high income Anglophone nations - generally most of the materials analysed fall within this cohort but not all. Additionally, many LMIC nations also regulate in the public interest but have different priorities and resourcing and framing of the public interest. Also relevant is whether the nation considers health care a public or private good. Many nations have or are working towards universal health systems recognising health as a public good - the US is an outlier in the OECD in its framing of health care as predominantly a private good.

AU: Thank you very much for pointing this out. We have revised the text to reflect the point around LMIC (beginning on line 75) and have tried to nuance the discussion around the framing of health as a public or private good throughout the manuscript (changes highlighted).

3. Line 223 while the authors note that most of the literature did not focus on the public interest. I wonder if this a framing issue rather than a lack of focus as, as noted by the authors, this was implicit rather than explicit. Thus I wonder whether this section should be focused on a deficit framing of not focusing on public interest or a more nuanced framing that considered implicitly? This would also be better aligned with the aforementioned amorphous nature of the public interest and how it might be quite jurisdictionally specific in its construction.

AU: We have changed the title of Finding 1 (page 12) to reflect your suggestion and have added a brief mention of the jurisdictional specificity to the findings. We have reviewed the rest of Finding 1 to ensure it aligns with the framing of this finding as ‘implicit consideration’ of the public interest.

4. Finding 3 (line 296). The literature included for this review was dominated by literature from the US - where competition and politics in health care are significant issues due to the political system of that country and I would suggest its framing of health as predominantly a private good and/or individual responsibility. Although there is a one sentence acknowledgement that competition might bear a different weight in the US versus other nations the same could be said for politics. In some nations the discussion is how to enable assessment for abortion, assisted dying etc to be undertaken virtually to enable access to what are clearly framed as health services (public goods) in those nations (an access argument). The generalisability of this finding as a whole, outside the US context, needs to be much more clearly and explicitly nuanced as part of the conclusion to this section

AU: We have reframed this finding to reflect your suggestions. Please see lines 333-335 and 351-353.

5. Finding 4 (line 341) The nature of cross-national reviews of legislation is complex. While issue 4 is a big one for federal states, there are unitary states (eg New Zealand etc) where regulation has always occurred nationally. For this group of countries cross-jurisdictional virtual practice is not a thing but regulating other aspects of the public interest around safety etc is the focus for any regulation around virtual care. This section needs to be nuanced to reflect this.

AU: We have revised Finding 4 to reflect this suggestion. Please see lines 365-369.

6. Line 452 please define regulatory sandbox and add a reference. It would be difficult for readers who lack knowledge of what this means and how and why it works to follow why this might be a beneficial approach in this context without further information.

AU: Per your suggestion, we have included a definition (with reference) of a regulatory sandbox beginning on line 472.

7. The conclusion and discussion section should also be nuanced per the comments above. While there is a clear statement of limitations in the conclusion these limitations and the lack of generalisability of some of the findings needs to be clearer throughout.

AU: We appreciate this suggestion and have tried to address this throughout the discussion and conclusion.

8. Just for your interest, another example of legal barriers to providing virtual care comes from Australia where currently discussions between health professionals and patients around assisted dying cannot be conducted over a carriage service due to federal law. See for example Del Villar, Katrine, Close, Eliana, Hews, Rachel, Willmott, Lindy, & White, Ben (2022) Voluntary assisted dying and the legality of using a telephone or internet service : The impact of Commonwealth ‘Carriage Service’ offences. Monash University Law Review, 47(1), pp. 125-173.

AU: Thank you very much for these references!

**REVIEWER #3:**

1. Abstract: the authors should include the sources of data under the methods section, even though they mentioned six multidisciplinary databases. The name of the database used to search the studies should be mentioned in the methods section.

AU: Thank you for the suggestion. We decided to ensure that the databases were described in the methods section of the manuscript since there were too many details to be captured in the short abstract. The names of the databases are included in the methods section under “Search Strategy” (lines 160-162).

2. Introduction: In the introduction of the study, the authors have very clearly explained the rationale and significance of this particular study. However, the introduction is a little bit long and it would be better if the authors reduced it one and a half pages.

AU: Thank you for the feedback. Both Reviewer 1 and Reviewer 2 requested further elaboration on certain points in the introduction, so we have tried to balance that with your suggestion to reduce the length. We have removed some sentences, amalgamated some sections, and removed the subheadings. With the addition of the other requested changes, the length is still longer than 1 ½ pages but we hope this is acceptable.

3. Methods: Authors should report in Table the keywords or search strings that they used to identify relevant studies during the search strategy.

AU: Thank you for the suggestion. This is available in the published protocol for the scoping review and we have clarified that an example database search (MEDLINE) with all search terms and string searches can be found with this publication (see lines 158-159). If the editors suggest (and it does not constitute duplicate publishing), we can also include this search as a supplementary file to this manuscript.
